# Acupuncture for the treatment of diabetic peripheral neuropathy in the elderly: a systematic review and meta-analysis

**DOI:** 10.3389/fmed.2024.1339747

**Published:** 2024-06-14

**Authors:** Xinyu Zhang, Lingyong Xiao, Yuan Qin, Huan Yang, Xiangcheng Wei, Lanping Li, Shiqing Zhao, Xiaoyu Dai

**Affiliations:** ^1^Department of Acupuncture and Moxibustion, First Teaching Hospital of Tianjin University of Traditional Chinese Medicine, Tianjin, China; ^2^National Clinical Research Center for Chinese Medicine Acupuncture and Moxibustion, Tianjin, China

**Keywords:** acupuncture, diabetic peripheral neuropathy, the elderly, meta-analysis, systematic review

## Abstract

**Background:**

Diabetic peripheral neuropathy (DPN) is one of the most common complications of diabetes mellitus, often causing pain or numbness in the patient’s limbs and even leading to amputation and death. Elderly patients with DPN usually have higher morbidity and more severe results. Acupuncture has been widely used as an effective treatment for DPN in China. However, the efficacy of acupuncture in the treatment of DPN remains unclear. In this review, we aimed to explore the impact of acupuncture in alleviating symptoms of DPN.

**Method and analysis:**

Six databases were searched from inception to October 2023. We searched Medline, EMBASE, Cochrane Central Register of Controlled Trials (CENTRAL), and three Chinese databases, namely China National Knowledge Infrastructure (CNKI), SinoMed, and Wanfang. All randomized controlled trials related to the effect of acupuncture on DPN will be included. There was no restriction in language or publication year. The primary outcome is the response rate. The secondary outcomes are the Toronto clinical scoring system (TCSS), nerve conduction velocities (NCVs), and blood glucose before and after the treatment. Two researchers will be responsible for the selection of study, data extraction, and assessment of study quality independently. RevMan V5.1.0 software will be used to assess the risk of bias and generate data.

**Results:**

We searched 4518 studies, among which 9 RCTs were considered eligible. Overall, acupuncture treatment had a higher response rate than controls (relative risk (RR), −2.87 [95% confidence interval (CI), −5.27 to −0.48], *p* = 0.02) and significantly alleviated the symptoms of DPN patients, reduced their blood glucose levels, and improved their NCVs compared to the control group. This study will provide a high-quality synthesis of current available evidence for the clinical treatment of DPN with this therapy.

**Conclusion:**

The results suggested that acupuncture might be effective in improving symptoms of DPN in elderly patients. Owing to the overall low quality of the literature included, we need more large-sample, high-quality, and low-bias studies to prove it.

## 1 Introduction

Diabetic peripheral neuropathy (DPN) is one of the most common complications of diabetes mellitus (DM). DPN is characterized by the functional loss of cutaneous receptors and proprioceptive sensation (1). Its typical symptoms include numbness and pain, which start most often in the feet and lower legs of DM patients. Up to 50% of diabetic peripheral neuropathies are asymptomatic, which is often missed until the disease progresses further, at which point it is almost irreversible, and should be treated with prompt preventive care (2). In addition, it is a leading cause of lower limb amputation and disabling neuropathic pain, which has a disastrous effect on the quality of life of patients and even leads to a shortened life expectancy (only 2 years on average) (3).

The incidence of DPN among newly diagnosed diabetic patients is 29.4% (4). Age is an independent risk factor for DPN. The incidence of DPN increased significantly with each 10-year increase in age, and diabetic patients older than 60 years of age were significantly associated with the incidence of DPN (4). In addition, older people with DPN experience higher fall risks compared to healthy older people, which can lead to serious consequences. With the progress of global population aging, the number of elderly patients with DPN has further increased, highlighting the need to address this issue in elderly populations (5).

At present, there is still a lack of treatment that targets underlying nerve damage. Prevention and early intervention are the key measures in the care of DPN (2). Appropriate interventions can reduce ulcers by 60% and amputations by 85% in those with high-risk diabetic neuropat (6). Based on the special characteristics of elderly patients with DPN, long-term use of drugs may increase the risk of adverse reactions (7). Therefore, clinical attention is gradually focused on the application of non-pharmacological treatment of elderly patients with DPNs. Acupuncture, a complementary and alternative therapy based on the meridian theory of traditional Chinese medicine, is currently widely used in China for the treatment of DPN. According to reports in the literature, acupuncture is an effective method for treating DPN, can improve nerve conduction velocities (NCVs) and clinical symptoms, and can slow down the development of DPN (8). We conducted a systematic review and meta-analysis to further explore the relationship between acupuncture and DPN.

## 2 Method

### 2.1 Literature search

This review was reported following the principles of the Preferred Reporting Items for Systematic Review and Meta-Analyses (PRISMA) (9). We searched Medline, EMBASE, Cochrane Central Register of Controlled Trials (CENTRAL), and three Chinese databases, namely China National Knowledge Infrastructure (CNKI), SinoMed, and Wanfang, from inception to October 2023. There was no restriction in language or publication year. The search strategy of this study is shown in Supplementary material 1. The flow diagram of the study selection process is shown in Supplementary material 3.

### 2.2 Study selection and data extraction

We considered RCTs concerning the efficacy of acupuncture treatment of DPN in patients 60 years of age or older. The intervention was acupuncture treatment. Due to the current lack of consensus and uniform standards on the definition of acupuncture, we adopt the International Organization for Standardization (ISO)'s definition of acupuncture: Acupuncture therapy refers to the entire process of inserting acupuncture needles into the body and applying appropriate maneuvers after the insertion, which involves the healthcare provider’s method of selecting acupuncture points (10), including electro-acupuncture, scalp acupuncture, warm acupuncture, fire acupuncture, and needle knife. The control group was pharmacotherapy, other non-pharmacotherapy, or invalid groups. The trial group can be included if it consists of acupuncture and control group therapy. This article excluded studies that compared different types of acupuncture and reported only one single outcome indicator. If additional information or data are required, we will contact the authors of the study.

Two independent reviewers (Huan Yang and Yuan Qin) conducted literature searches separately, initially screened titles and abstracts according to the requirements, and included qualified articles after reading the full text. In case of disagreement, the third reviewer will make the final decision. The two investigators independently extracted data from the eligible literature into the Microsoft Excel spreadsheet and extracted the following data according to the predesigned forms: first author name, publication year, country, study design, participant characteristics, intervention indication, male-to-female ratio, duration of treatment, outcome measures, and adverse events.

### 2.3 Outcome assessment

The primary endpoint was the response rate. The secondary endpoints included the Toronto clinical scoring system (TCSS), NCVs (median nerve sensory nerve conduction velocity (SNCV), common peroneal nerve MNCV, and common peroneal nerve SNCV), and blood glucose (fasting glucose and glycosylated hemoglobin). The outcome of the patient was divided into three categories-significant effective (no abnormality in neurological examination and disappearance of subjective symptoms), effective (improvement in neurological examination and alleviated subjective symptoms), and ineffective (no improvement in neurological examination and subjective symptoms), and then calculate the response rate: the response rate = significant effective rate + effective rate.

### 2.4 Risk of bias assessment

The included randomized controlled trials were independently assessed by two evaluators (Huan Yang and Yuan Qin) according to the Cochrane Risk of Bias Assessment Tool. The following items were evaluated: random sequence generation, allocation concealment, blinding of participants and personnel, blinding of outcome assessment, incomplete outcome data, selective reporting, and other biases. The overall risk of bias for each study was summarized after evaluating them on three levels: low risk, high risk, and unclear risk. Disagreements were resolved by negotiation, and if no consensus could be reached, the decision was made by a third evaluator.

### 2.5 Statistical analysis

Statistical analysis was carried out using Review Manage software (V5.1.0, Nordic Cochrane Center, Copenhagen, Denmark). Dichotomous data were presented as relative risk (RR) and continuous data as mean difference (MD) and 95% confidence interval (95%CI). The major assumption of a fixed-effect model is that all effect sizes share a common mean, and thus that variation among data is solely attributable to sampling error (11). This assumption, however, is unrealistic for most meta-analyses; the random-effects model will be used throughout this article.

Heterogeneity among studies was tested by Q-test and *I*^2^-test statistics. If its *p*-value >0.05 indicates no significant heterogeneity, the differences between the studies are caused by random factors. If the *p*-value is <0.05, it means significant heterogeneity that the differences between the studies are not random, but rather due to a factor that causes heterogeneity between cases.

## 3 Result

### 3.1 Study description

A total of 4158 studies were retrieved in this study; 2513 duplicate studies were screened out, and 1309 studies irrelevant to this study were excluded after further reading of the title and abstract. The complete text was then read, and 327 unqualified studies were screened out according to the inclusion and exclusion criteria of the literature. Nine studies (12–20) were finally included for meta-analysis, and the diagram of the screening process is shown in Supplementary material 3. The included studies were all in Chinese, had a sample size of 751 patients (383 patients in the experimental group and 368 in the control group), treatment durations ranging from 10 days to 3 months, no distinction between type 1 and type 2 DM, and no follow-up. Of the nine studies, all patients were treated with conventional Western medicine, except for one study (19) in the control group, which used walking ladder training (WLT). Except for acupuncture with specimen matching points (20) in one RCT in the experimental group and warm acupuncture (19) in one RCT, millineed needling was used in the other seven studies. The basic characteristics of the included studies are shown in Table 1 and Supplementary material 2.

**Table 1:** Characteristics of the included trials.

Study ID	N (T/C)	Age (T/C, years)	Acupuncture intervention	Control intervention	Outcome
Ye xin2020	93 (46/47)	72.13 ± 4.25/72.92 ± 3.73	Specimen matching points acupuncture	Therapeutics	①③④⑤⑥⑦⑧⑨
Chen Hualu2022	119 (60/59)	66.62 ± 5.17/65.71 ± 4.28	Hand and foot warm acupuncture+walking step training	Mecobalamin	①②⑩⑪⑫⑬
Li Lihong2015	30 (15/15)	72 ± 5.82	Acupuncture+Mecobalamin	walking step training	①②⑭
Yu Shaoqing2017	90 (45/45)	60–69/60–68	Acupuncture+Mecobalamin	Mecobalamin	①②⑭
Han Qing2018	64 (34/30)	6.9 ± 3.6/5.6 ± 4.7	Acupuncture+Mecobalamin	Mecobalamin	①②④⑥
Xie Aixian2017	100 (48/52)	70.6 ± 3.2	Acupuncture+DL-Thioctic acid	Mecobalamin	①⑤⑥⑮⑯
Wang Zichun2013	82 (41/41)	77.5 ± 4.3/81.2 ± 2.1	Acupuncture	Mecobalamin+Vitamin B1	①③④⑤⑥⑰⑱
Feng Xiao2018	92 (46/46)	71.5 ± 4.6/71.6 ± 4.7	Acupuncture	Sham-acupuncture	①②
Yu Haoyan2017	81 (43/38)	70.67 ± 4./70.44 ± 4.32	Acupuncture	Sham-acupuncture	⑦⑲

① The response rate, ② the Toronto clinical scoring system (TCSS), ③ median nerve MNCV, ④ median nerve SNCV, ⑤ common peroneal nerve MNCV, ⑥ common peroneal nerve SNCV, ⑦ blood glucose, ⑧ blood lipid, ⑨ inflammatory cytokines, ⑩ Michigan Diabetes Neuropathy Score (MDNS), ⑪ tibial nerve MNCV, ⑫ tibial nerve SNCV, ⑬ hemodynamics, ⑭ pain relief effect, ⑮ Michigan Neurological Screening Inventory (MNSI), ⑯ physical examination score, ⑰ ulnar nerve MNCV, ⑱ ulnar nerve SNCV, and ⑲ treatment satisfaction.

### 3.2 Assessment of quality and bias

We used the Cochrane Risk of Bias Assessment Tool to assess the quality of the included papers. Nine studies described the specific randomization scheme in detail with allocation concealment, six studies used random digitization tables (low risk), one study used computerized randomization (low risk), one study used coin-flip randomization (low risk), and one study used an incorrect randomization scheme based on the order of treatment (high risk). None of the nine studies explicitly said whether or not they were blinded (unclear risk). Nine studies had complete outcome data (low risk). In the reporting of outcome selectivity, one study did not define and describe outcomes in advance, and there was a possibility of reporting bias (high risk). Among other biases, it was unclear whether there were other biases in the literature, except for one where the sample size was too small (high risk). The risk of bias in included studies is shown in Supplementary material 4.

### 3.3 The response rate

**Figure 1:**
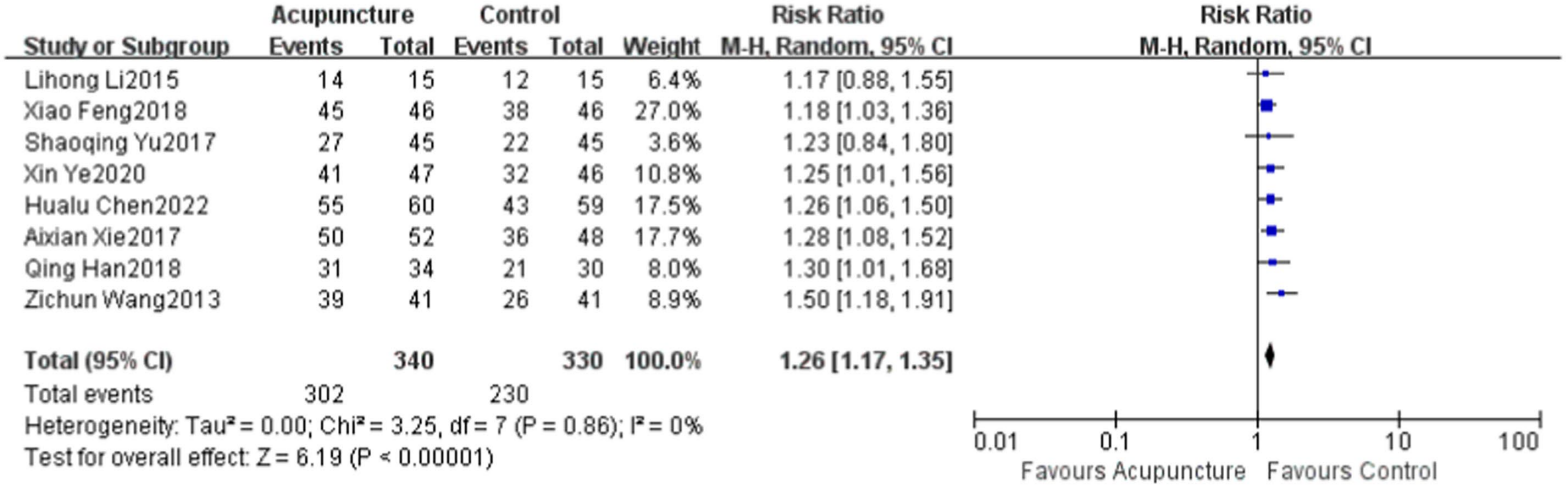
The Response rate to acupuncture compared to the control group in eight studies. CI, confidence interval; df, degree of freedom; M–H, Mantel–Haenszel test.

A total of eight (12–17, 19, 20) studies evaluated the clinical response rate in the literature, with a total of 670 patients included, and there was no significant heterogeneity among the studies (*p* = 0.86, *I*^2^ = 0), which was evaluated using a random-effects model. For the treatment of DPN, the response rate of the experimental group was higher than that of the control group (RR = 4.49, 95% CI: 1.26 [1.17,1.35], *Z* = 7.49, *p* < 0.00001) (Figure 1).

### 3.4 The Toronto clinical scoring system (TCSS)

**Figure 2:**
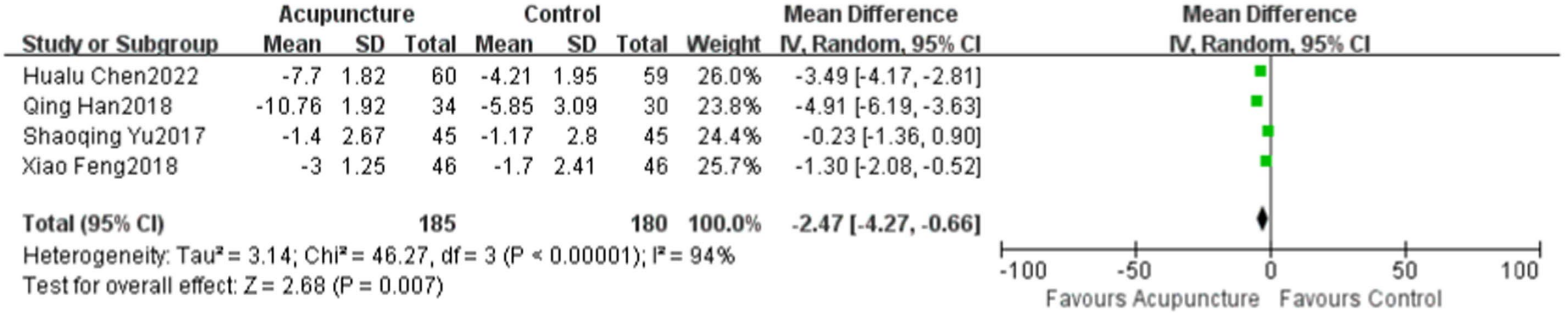
The Score of the TCSS compared to the control group in four studies.

A total of four papers (13, 14, 17, 19) evaluated the TCSS. One article did not report pre-treatment data but only reported post-treatment outcomes, and we have not yet contacted the authors to obtain the original data; therefore, this article was excluded. A total of 319 patients were included, and the studies were significantly heterogeneous from each other (*p* < 0.00001, *I*^2^ = 94%), and were evaluated using a random-effects model. For the treatment of DPN, the TCSS score of the experimental group was better than that of the control group, and the difference was statistically significant (MD = −2.87, 95% CI: −5.27 to −0.48, *Z* = 2.35, *p* = 0.02) (Figure 2).

### 3.5 Nerve conduction velocities (NCVs)

**Figure 3:**
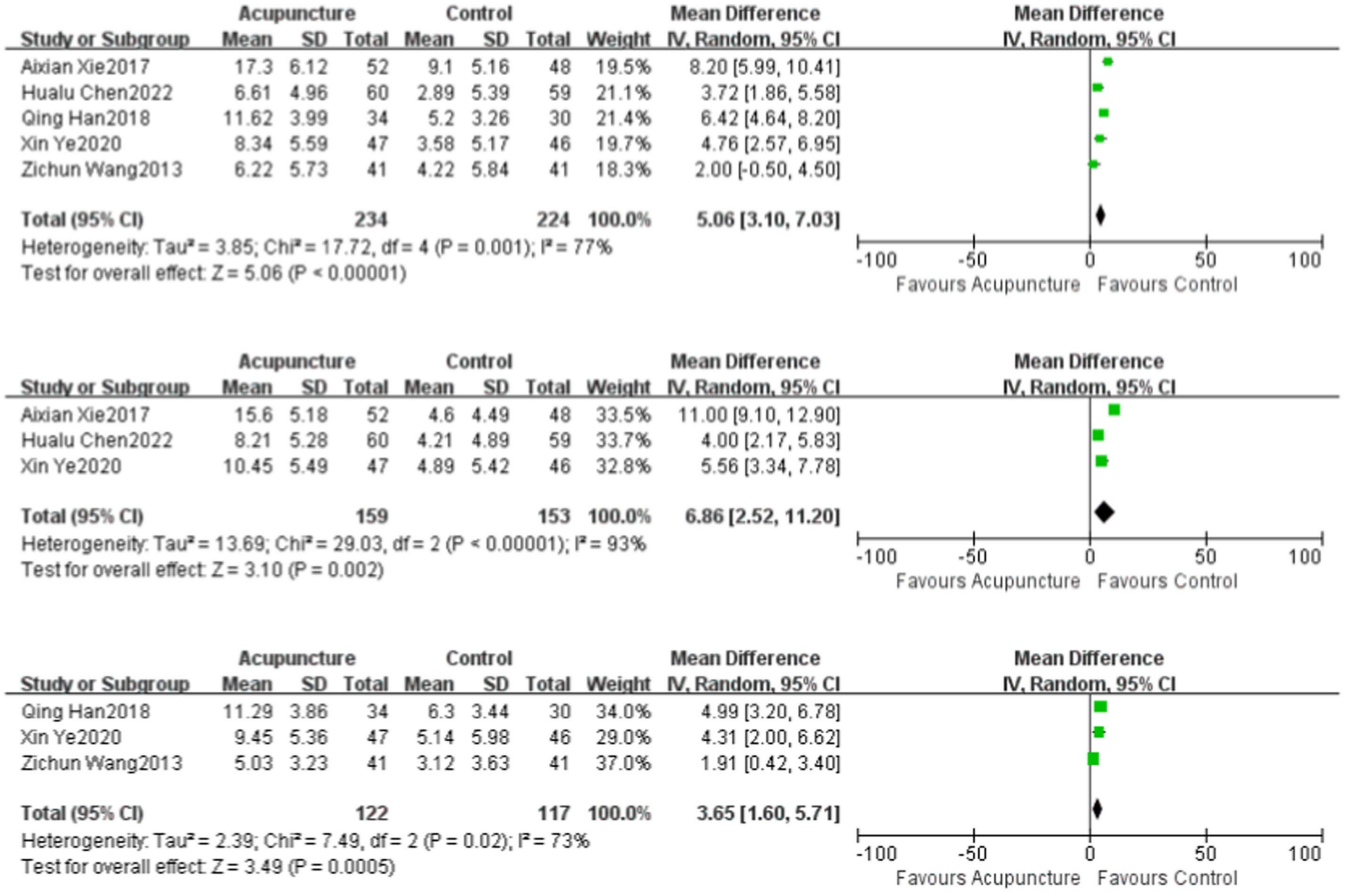
The Velocities of common peroneal nerve SNCV, the common peroneal nerve MNCV, and median nerve SNCV compared to the control group.

Three studies (14, 16, 20) in the literature evaluated median nerve SNCV and included a total of 239 patients. Three studies (15, 19, 20) evaluated the common peroneal nerve MNCV and included a total of 312 patients. Five studies (14–16, 19, 20) reported the common peroneal nerve SNCV and included a total of 458 patients. All had significant heterogeneity: median nerve SNCV (*p* = 0.02, *I*^2^ = 73%), common peroneal nerve MNCV (*p* < 0.00001, I^2^ = 93%), and common peroneal nerve SNCV (*p* = 0.001, *I*^2^ = 77%). A random-effects model was used for all. For the treatment of DPN, the improvements in motor NCVs of the experimental group were better than those of the control group, and the differences were statistically significant: median nerve SNCV (MD = 3.65, 95% CI: 1.60 to 5.71, *Z* = 3.45, *p* = 0.0005), common peroneal nerve MNCV (MD = 6.86, 95% CI: 2.52 to 11.2, Z = 3.10, *p* = 0.002), and common peroneal nerve SNCV (MD = 5.06, 95% CI: 3.10 to 7.03, *Z* = 5.06, *p* < 0.00001). Acupuncture effectively improved motor NCVs (Figure 3).

### 3.6 Blood glucose

Two studies (18, 20) evaluated fasting blood glucose and glycosylated hemoglobin in the literature, and a total of 174 patients were included. There was significant heterogeneity among the studies on fasting blood glucose (*p* = 0.0006, *I*^2^ = 92%) and glycosylated hemoglobin (*p* < 0.00001, *I*^2^ = 96%), which were evaluated using a random-effects model. For the treatment of DPN, the experimental group showed better improvement in fasting blood glucose and glycosylated hemoglobin than the control group, and the difference was statistically significant. The fasting blood glucose of the experimental group was lower than that of the control group (MD = −1.2, 95% CI: −2.34 ~ −0.07, *Z* = 2.08, *p* = 0.04). The HBA1c of the experimental group was lower than that of the control group (MD = −1.45, 95% CI: −2.69 ~−0.21, *Z* = 2.28, *p* = 0.02).

**Figure 4:**
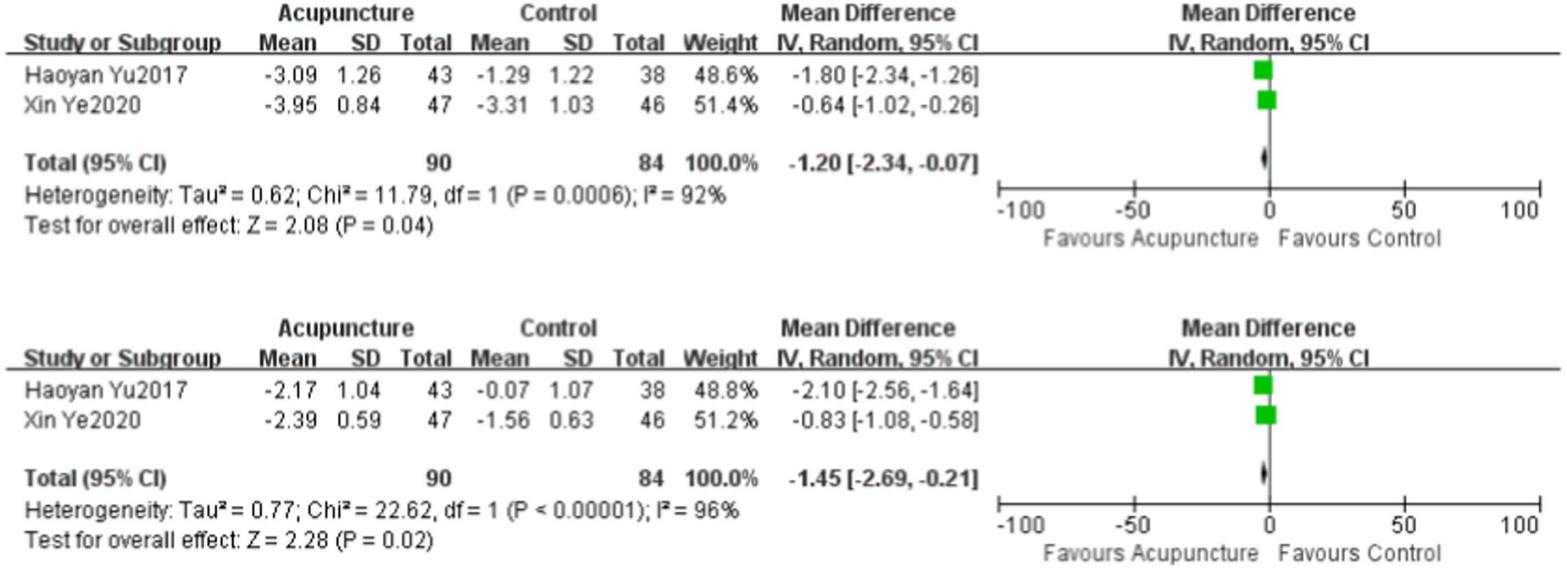
The Level of FPG and HbAlc compared to the control group.

## 4 Conclusion

We managed to minimize the risk of bias and draw as objective conclusions as possible by formulating a detailed protocol in advance, conducting a comprehensive search for published trials, using clear findings, data extraction, and data analysis methods, and rigorously performing literature quality assessments. In conclusion, acupuncture treatment is significantly better than regular treatment and can effectively alleviate the symptoms of DPN patients, reduce their blood glucose levels, and improve their NCVs. The results of this study have a certain degree of reliability; however, due to the above shortcomings and limitations, more large-sample, high-quality, and low-bias studies are needed to prove it.

## 5 Discussion

This review focuses on assessing the efficacy of acupuncture in treating elderly patients with DPN. A total of nine randomized controlled trials were included, with acupuncture, specimen matching acupuncture, and warm acupuncture as the intervention group and methylcobalamin, vitamin B, diabetes education, and walking step training as the control group.

Nine studies with a total of 751 randomized participants were eligible for inclusion in this review. For the main outcome indicators, acupuncture alleviated the symptoms of DPN in the elderly compared to conventional Western medical treatment. Four studies evaluated the efficacy of acupuncture in treating DPN using the TCSS, with significant differences. The results of this meta-analysis showed a significant increase in the response rate and a significant decrease in the TCSS scores of patients with acupuncture intervention. The response rate and TCSS can be assessed comprehensively in DPN patients and can show visually that acupuncture relieves the clinical symptoms of the patients. During the pathogenesis of elderly patients with DPN, hyperglycemia activates the glucose polyol pathway and generates a large number of free radicals that damage nerves, leading to a decrease in MNCV and SNCV (21). Three studies evaluated median nerve SNCV, three studies evaluated common peroneal nerve MNCV, and five studies reported common peroneal nerve SNCV with statistically significant changes in NCVs. The NCVs are an important outcome indicator of neurologic function, but they cannot be used as direct evidence of the clinical efficacy of DPN. The study also showed that acupuncture can improve NCV and control blood glucose concentration in patients. Therefore, acupuncture can not only alleviate clinical symptoms in DPN patients but also control blood glucose and repair nerves, improving clinical efficacy.

We found that the acupuncture group had a lower incidence of adverse events and dropout rates. As a non-pharmacological treatment, acupuncture therapy will be a safe complementary approach to treating elderly patients with DPN. However, the evidence has a low level of certainty because of the small number of included studies and the fact that some of the studies did not report the incidence of adverse events or dropout rates. None of the literature included in this study followed up with patients to compare the long-term efficacy of acupuncture in the treatment of DPN in the elderly.

DPN patients’ blood is mainly hypercoagulable, which can easily lead to tissue ischemia and hypoxia (22). In addition, in patients with long-term high glucose levels, coagulation and anticoagulation factors are expressed abnormally, resulting in dyslipidemia and accelerated vascular lesions, both of which are associated with DPN disease progression (23). In addition, Tang et al. (24) found that the possible mechanism of alleviating the symptoms of DPN by acupuncture is related to the regulation of P2X4 expression and inflammatory response in rat spinal microglia. An RCT (20) shows that acupuncture can effectively reduce lipid concentration and improve inflammatory cytokines. Hualu Chen et al. (19) show that acupuncture can effectively reduce whole blood viscosity, plasma-specific viscosity, and fibrinogen levels while also improving blood condition, thereby accelerating blood microcirculation, improving local nutritional status, and alleviating patients’ clinical symptoms. This suggests that acupuncture may improve the symptoms of elderly patients with DPN by improving blood rheology, inhibiting inflammatory factors, and increasing nerve conduction speed, which will provide some inspiration for future exploration of the DPN mechanism. To some extent, this study showed that acupuncture could improve the symptoms of patients by reducing blood glucose and increasing NCV, but the discussion of blood rheology, lipid concentration, and inflammatory factors was not involved. Some studies have shown that the decline in inflammatory factors and lipid markers can delay the progression of DPN disease, providing new treatment ideas for DPN patients.

The meta-analysis showed that except for the homogeneity of the total effective rate, the heterogeneity of the other indicators was high, which may be due to the following reasons: ① none of the included studies mentioned DPN caused by type I or type II DM, and their specific pathogenesis may be different; ② the disease duration of the patients with DPN included in the study was different, and some of the studies did not describe it in detail, which resulted in the difference in therapeutic efficacy; ③the regular treatment of Western medicines used was not the same, and the dosage also varied, which may cause different therapeutic effects; and ④ the acupoints, manipulation, and period of treatment used in the nine studies are not the same, thus affecting the therapeutic effects.

There are several potential limitations to the current study. First, only nine randomized controlled trials were included in this study, which is a small sample size. The low quality of the literature included in this paper and the small sample size reduce the credibility of the evidence, second, although a comprehensive search was conducted, publication and language bias may be present. Third, the effects of acupuncture on DPN at different acupoints and stimulation volume pairs have not been demonstrated, and nine of the nine randomized controlled trials followed up, so the long-term efficacy of acupuncture for DPN is unclear. Fourth, none of the included literature mentioned blinding, resulting in a low overall quality of the literature. In addition, some of the included studies did not indicate the duration and staging of the patients, thus affecting the accuracy of the baseline. Because there is no standardized protocol for the treatment of DPN, there are no specific standards for the acupoints selection, manipulation, and duration of treatment used in clinical practice, and the methods of measurement are inconsistent. Future studies should aim to address these limitations, increase the number of sample sizes, improve the quality of clinical evidence, and explore the optimal dose and duration of acupuncture for DPN.

## Data availability statement

The original contributions presented in the study are included in the article/Supplementary material, further inquiries can be directed to the corresponding author.

## Author contributions

XZ: Writing – original draft, Writing – review & editing, Formal analysis, Methodology. LX: Writing – original draft, Writing – review & editing, Formal analysis, Methodology. YQ: Data curation, Writing – original draft. HY: Data curation, Writing – original draft. XW: Writing – original draft, Data curation. LL: Writing – original draft, Data curation. SZ: Writing – original draft, Data curation. XD: Formal analysis, Funding acquisition, Writing – original draft, Writing – review & editing, Supervision.
