# Supplementary material for: Acupuncture for the treatment of diabetic peripheral neuropathy in the elderly: a systematic review and meta-analysis
Source: Front Med (Lausanne). 2024 Jun 14;11:1339747. doi: 10.3389/fmed.2024.1339747 (PMC11211378; doi:10.3389/fmed.2024.1339747)
Supplement: Supplementary file 1 [file Data_Sheet_1.docx]

**Supplementary Online Content**

**Supplementary material 1.** Search strategy for Medline

**Supplementary material 2.** Basic information about included studies

**Supplementary material 3.** The flow diagram of the study selection process

**Supplementary material 4.** Risk of bias in included studies

Supplementary material 1. Search strategy for Medline

| #1 | Randomized Controlled Trial[MeSH] |
| --- | --- |
| #2 | Controlled Clinical Trial[pt] |
| #3 | Randomized Controlled Trial[pt] |
| #4 | Controlled clinical trial[Title/Abstract] |
| #5 | Randomized Controlled Trial[Title/Abstract] |
| #6 | Controlled Clinical Trial[Title/Abstract] |
| #7 | Controlled clinical trial[Title/Abstract] |
| #8 | Randomized [Title/Abstract] |
| #9 | Randomised [Title/Abstract] |
| #10 | RCT[Title/Abstract] |
| #11 | placebo*[Title/Abstract] |
| #12 | sham[Title/Abstract] |
| #13 | group[Title/Abstract] |
| #14 | trial[Title/Abstract] |
| #15 | or/#2-#14 |
| #16 | #1or#15 |
| #17 | Acupuncture Therapy[MeSH] |
| #18 | Acupuncture [MeSH] |
| #19 | #17or#18 |
| #20 | Acupuncture Therapy[Title/Abstract] |
| #21 | Acupuncture[Title/Abstract] |
| #22 | Ear acupuncture[Title/Abstract] |
| #23 | Auricular acupuncture [Title/Abstract] |
| #24 | Auricular needle [Title/Abstract] |
| #25 | Warm acupuncture [Title/Abstract] |
| #26 | Warm needle [Title/Abstract] |
| #27 | Fire needle[Title/Abstract] |
| #28 | Electropuncture [Title/Abstract] |
| #29 | Electroacupuncture [Title/Abstract] |
| #30 | Electro-acupuncture [Title/Abstract] |
| #31 | or/#20-#30 |
| #32 | #19or#31 |
| #33 | Diabetic Neuropathies [MeSH] |
| #34 | DPN[Title/Abstract] |
| #35 | Diabetic peripheral neuropathy[Title/Abstract] |
| #36 | Diabetic peripheral neuropathies[Title/Abstract] |
| #37 | Diabetic Neuropathy[Title/Abstract] |
| #38 | Diabetic Neuropathies[Title/Abstract] |
| #39 | Diabetic Neuralgia[Title/Abstract] |
| #40 | Diabetic Neuralgias[Title/Abstract] |
| #41 | Painful Diabetic Neuropathies[Title/Abstract] |
| #42 | Painful Diabetic Neuropathy[Title/Abstract] |
| #43 | Symmetric Diabetic Proximal Motor Neuropathy[Title/Abstract] |
| #44 | Asymmetric Diabetic Proximal Motor Neuropathy[Title/Abstract] |
| #45 | Diabetic Asymmetric Polyneuropathy[Title/Abstract] |
| #46 | Diabetic Asymmetric Polyneuropathies[Title/Abstract] |
| #47 | Diabetic Mononeuropathy[Title/Abstract] |
| #48 | Diabetic Mononeuropathies[Title/Abstract] |
| #49 | Diabetic Mononeuropathy Simplex[Title/Abstract] |
| #50 | Diabetic Mononeuropathy Simplices[Title/Abstract] |
| #51 | Diabetic Polyneuropathy[Title/Abstract] |
| #52 | Diabetic Polyneuropathies[Title/Abstract] |
| #53 | or/#34-#52 |
| #54 | #32or#53 |

This search strategy was modified to be suitable for other electronic databases

**Supplementary material 2 basic information about included studies**

| **Study**  **ID** | **Treatment modality** | | | | **Sample sizes** | | **Sex(male)** | | **Age** | | **Course of disease** | |
| --- | --- | --- | --- | --- | --- | --- | --- | --- | --- | --- | --- | --- |
|  | **Experimental group** | | **Control group** | |  |  |  |  |  |  |  |  |
|  | **Therapeutic** | **Numb**  **er of**  **times** | **Therapeutics** | **Numb**  **er of**  **times** | **Experim**  **ental**  **group** | **Contr**  **ol**  **group** | **Experim**  **ental**  **group** | **Contr**  **ol**  **group** | **Experim**  **ental**  **group** | **Contr**  **ol**  **group** | **Experim**  **ental**  **group** | **Contr**  **ol**  **group** |
| Ye xin2020 | Specimen Matching Points" Acupuncture | 24Tim  es | Mecobalamin | 24Tim  es | 46 | 47 | 28 | 26 | 72.13±4.25 | 72.92±3.73 | 3.41±0.69 | 3.46±0.87 |
| Chen Hualu2022 | hand and foot warm acupuncture combined with walking step train  ing | 60Tim  es | walking step training | 36 Tim  es | 60 | 59 | 24 | 27 | 66.62±5.17 | 65.71±4.28 | 2.81±0.32 | 2.86±0.33 |
| Li Lihong2015 | Acupuncture  +  Mecobalamin | 45Tim  es | Mecobalamin | 28Tim  es | 15 | 15 | NA | NA | 72±5.82 | | **NA** | **NA** |
| Yu Shaoqing2017 | Acupuncture  +  Mecobalamin | NA | Mecobalamin | NA | 45 | 45 | 25 | 21 | 60-69 | 60-68 | 3-5.6 | 3-5 |
| Han Qing2018 | Acupuncture  +  Mecobalamin | 20Tim  es | Mecobalamin | 20Tim  es | 34 | 30 | 22 | 19 | 6．9±3．6 | 5．6±4．7 | 0.5-6.5 | 0.55-7 |
| Xie Aixian2017 | Acupuncture  +  DL-Thioctic acid | 14Tim  es | Mecobalamin+Vitamin B1 | 14Tim  es | 48 | 52 | NA | NA | 70.6±3.2 | | NA | NA |
| Wang Zichun2013  7 | Acupuncture | 18Tim  es | Sham-acupun  cture | 18Tim  es | 41 | 41 | 21 | 17 | 77.5±4.3 | 81.2±2.1 | NA | NA |
| Feng Xiao2018 | Acupuncture | 42Tim  es | Sham-acupun  cture | 42Tim  es | 46 | 46 | 24 | 23 | 71.5±4.6 | 71.6±4.7 | NA | NA |
| Yu Haoyan2017 | Acupuncture | 10Tim  es | Sham-acupun  cture | 10Tim  es | 43 | 38 | 26 | 22 | 70.67±4. | 70.44±4.32 | NA | NA |

**Supplementary material 3** The flow diagram of the study selection process**
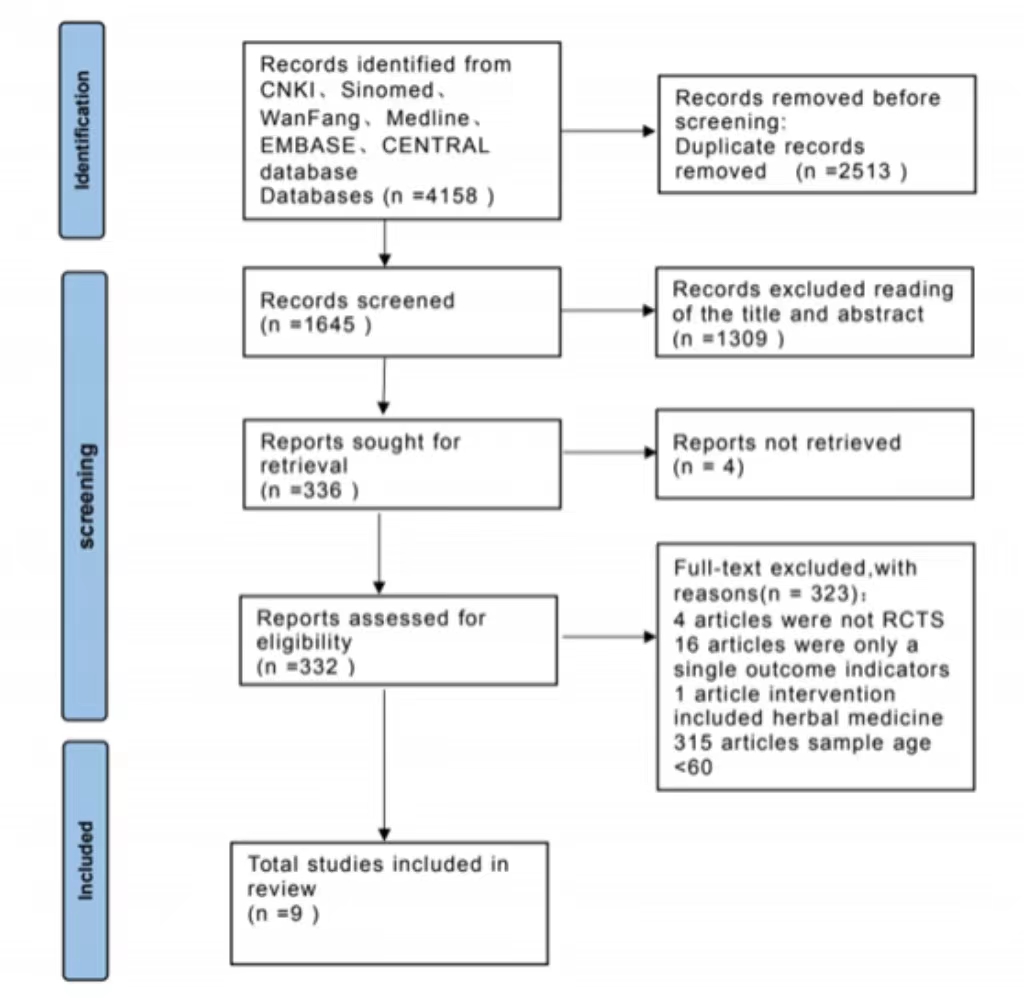
**


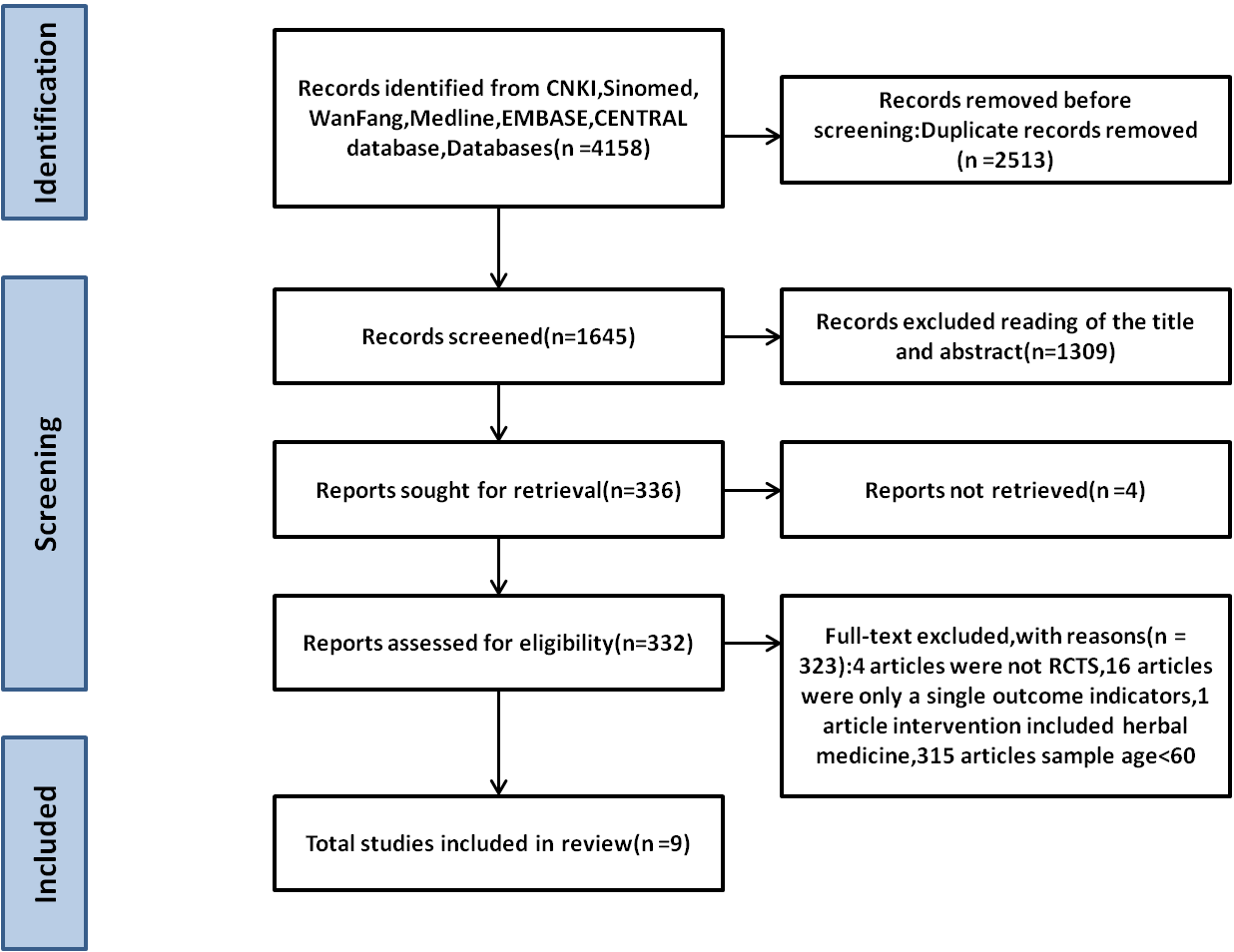


**Supplementary material 4.** Risk of bias in included studies


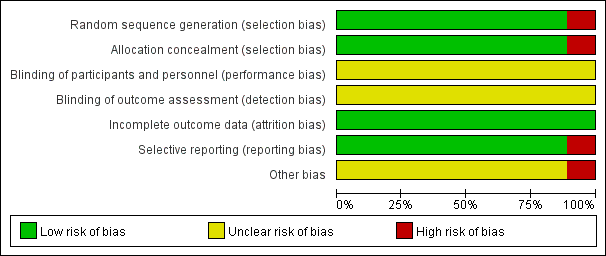


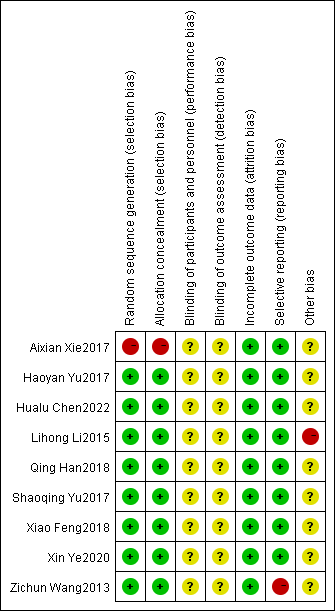


Note: Green represents low risk of bias, yellow represents unclear, and red represents high risk of bias.
